# Burst nucleation by hot injection for size controlled synthesis of *ε*-cobalt nanoparticles

**DOI:** 10.1186/s13065-016-0156-1

**Published:** 2016-03-08

**Authors:** Eirini Zacharaki, Maria Kalyva, Helmer Fjellvåg, Anja Olafsen Sjåstad

**Affiliations:** Department of Chemistry, Centre for Materials Science and Nanotechnology, University of Oslo, Blindern, P.O. Box 1033, 0315 Oslo, Norway; Department of Chemistry, inGAP Centre of Research-based Innovation, University of Oslo, Blindern, P.O. Box 1033, 0315 Oslo, Norway

**Keywords:** *ε*-Cobalt nanoparticles, Hot injection synthesis, Particle size control, Reproducibility

## Abstract

**Background:**

Reproducible growth of narrow size distributed *ε*-Co nanoparticles with a specific size requires full understanding and identification of the role of essential synthesis parameters for the applied synthesis method. For the hot injection methodology, a significant discrepancy with respect to obtained sizes and applied reaction conditions is reported. Currently, a systematic investigation controlling key synthesis parameters as injection-temperature and time, metal to surfactant ratio and reaction holding time in terms of their impact on mean ($\bar{D}$_mean_) and median ($\bar{D}$_median_) particle diameter using dichlorobenzene (DCB), Co_2_(CO)_8_ and oleic acid (OA) as the reactant matrix is lacking.

**Methods:**

A series of solution-based *ε*-Co nanoparticles were synthesized using the hot injection method. Suspensions and obtained particles were analyzed by DLS, ICP-OES, (synchrotron)XRD and TEM. Rietveld refinements were used for structural analysis. Mean ($\bar{D}$_mean_) and median ($\bar{D}$_median_) particle diameters were calculated with basis in measurements of 250–500 particles for each synthesis. 95 % bias corrected confidence intervals using bootstrapping were calculated for syntheses with three or four replicas.

**Results:**

*ε*-Co NPs in the size range ~4–10 nm with a narrow size distribution are obtained via the hot injection method, using OA as the sole surfactant. Typically the synthesis yield is ~75 %, and the particles form stable colloidal solutions when redispersed in hexane. Reproducibility of the adopted synthesis procedure on replicate syntheses was confirmed. We describe in detail the effects of essential synthesis parameters, such as injection-temperature and time, metal to surfactant ratio and reaction holding time in terms of their impact on mean ($\bar{D}$_mean_) and median ($\bar{D}$_median_) particle diameter.

**Conclusions:**

The described synthesis procedure towards *ε*-Co nanoparticles (NPs) is concluded to be robust when controlling key synthesis parameters, giving targeted particle diameters with a narrow size distribution. We have identified two major synthesis parameters which control particle size, *i.e*., the metal to surfactant molar ratio and the injection temperature of the hot OA–DCB solution into which the cobalt precursor is injected. By increasing the metal to surfactant molar ratio, the mean particle diameter of the *ε*-Co NPs has been found to increase. Furthermore, an increase in the injection temperature of the hot OA-DCB solution into which the cobalt precursor is injected, results in a decrease in the mean particle diameter of the *ε*-Co NPs, when the metal to surfactant molar ratio $\left( {\frac{[Co]}{[OA]}} \right)$ is fixed at ~12.9.

**Electronic supplementary material:**

The online version of this article (doi:10.1186/s13065-016-0156-1) contains supplementary material, which is available to authorized users.

## Background

Cobalt nanoparticles (NPs) are of importance due to applications linked to their magnetic and catalytic properties. Cobalt is a ferromagnetic metal and has size dependent properties at the nanoscale. During the last decades, magnetic cobalt NPs have been intensively investigated with respect to their use in data storage devices [1, 2] and sensors [3, 4] amongst others. Metallic cobalt nanoparticles are important catalysts in the conversion of synthesis gas to hydrocarbons, *i.e.* in the Fischer–Tropsch (FT) process. Typically, the catalysts used consist of Co NPs dispersed on an oxide support [5–7], prepared by impregnation, and followed by drying, calcination and activation steps. This way of preparation yields normally non-uniform Co NPs with respect to size and shape, which hinders the study of size-dependent catalytic properties. Systematic single parameter studies to correlate particle properties such as size, shape, atomic arrangement and chemical composition to magnetic behavior or catalytic performance, require highly refined and reproducible synthesis procedures. In addition, robust routes for deposition of the particles onto the support material are required [8].

Metallic cobalt crystallizes in hexagonal- and cubic close packed (hcp and ccp) structures, wherein the hcp variant is the stable modification below ~693 K [9]. In addition, metastable cobalt-variants are reported [10, 11]. Dinega and Bawendi [10] described *ε*-Co, with the *β*-Mn-type structure [12], crystallizing in space group *P*4_1_32 with 20 atoms in the unit cell. Notably, only solution based synthesis approaches give *ε*-Co NPs. The *ε*-Co phase transforms irreversibly during annealing in a non-oxidative atmosphere into hcp and ccp at ~573 and 773 K, respectively [1, 10, 13].

In the past decade, considerable progress has been made in the synthesis of monodispersed and well-defined cobalt NPs by colloidal chemical synthetic procedures [14]. The final product is colloidal Co NPs stabilized by surfactant molecules and dispersed in solvent media [1, 10, 15]. Studies by La Mer and Dinegar [16] show that a short burst of nucleation followed by slow diffusion controlled growth is critical to produce monodispersed particles [14, 17]. Dinega and Bawendi [10] synthesized and identified *ε*-Co in colloidal form by thermal decomposition of Co_2_(CO)_8_ in toluene in the presence of trioctylphosphine oxide (TOPO). The obtained colloidal particles were roughly spherical, with relative standard deviation (RSD) ~15 % and average diameter ~20 nm. Sun and Murray [1], as well as Puntes and Alivisatos [4] showed by using different synthetic conditions that neither Co_2_(CO)_8_ nor TOPO are essential for the formation of *ε*-Co. Recently, Iablokov et al. [18] obtained Co NPs in the sub 10 nm range using dichlorobenzene (DCB) as solvent, Co_2_(CO)_8_ as metal precursor and various surfactants. By using oleic acid (OA) as surfactant they explored the effect of injection temperature on particle size. They showed that the commonly used phosphorus containing surfactant TOPO results in phosphorus being present on the cobalt metal surface even after extensive catalyst pretreatment in a reductive atmosphere at elevated temperatures (*i.e.* 723 K). In their work TOPO was identified as a serious catalytic poison for CO_2_ hydrogenation [18]. Beside the work of Iablokov et al. [18] only Puntes et al. [19] and Ma et al. [20], have produced *ε*-Co NPs using OA as the sole surfactant with DCB as solvent and Co_2_(CO)_8_ as cobalt precursor, see Table 1. Ma et al. [20] have successfully produced *ε*-Co NPs over the 4–9 nm size range by varying the metal to surfactant molar ratio ($5 \le \frac{[Co]}{[OA]} \le 20$), while injecting the Co precursor in the hot OA-DCB solution at 463 K. In addition, Iablokov et al. [18] producted 3–10 nm *ε*-Co NPs by varying the temperature of the hot OA-DCB solution (441 ≤ T (K) ≤ 455). In their work, the metal to surfactant molar ratio was approximately 6.5. A significant discrepancy with respect to obtained sizes and applied reaction conditions can be noted. Presently the discrepancy between the studies is not understood and a systematic investigation using DCB, Co_2_(CO)_8_ and OA as the reactant matrix is lacking.

**Table 1:** Synthesis conditions of *ε*-Co NPs, using DCB-OA-Co_2_(CO)_8_

	DCB (mL)	Co_2_(CO)_8_ (mmol)	OA (mmol)	$\frac{[Co]}{[OA]}$	Reaction time (s)	Injection temperature (K)	$\bar{D}_{mean}$ (nm)	RSD (%)
Puntes [19]	18	1.6	0.63	5.0	300	455	N/A	10–20
Ma [20]	9	0.8	0.08	20.0	600	463	9^a^	N/A
	9	0.8	0.16	10.0	600	463	6^a^	N/A
	9	0.8	0.32	5.0	600	463	4^a^	N/A
Iablokov [18]	18	1.5	0.46	6.5	1200	455	3.2^a^	12.5
	18	1.5	0.46	6.5	1200	451	4.8^a^	6.3
	18	1.5	0.46	6.5	1200	447	6.8^a^	7.4
	18	1.5	0.46	6.5	1200	<441	10.2^a^	5.9
Present study	18	1.5	0.24	12.9	1800	452	4.6^a^	19.6
	18	1.5	0.24	12.9	1800	447	7.1^a^	14.3
	18	1.5	0.24	12.9	1800	443	7.9^a^	17.5
	18	1.5	0.24	12.9	1800	441	9.6^a^	14.0
	18	1.5	0.24	12.9	1800	437	9.4^a^	15.5
	18	1.5	1.45	2.1	1800	441	2^b^	
	18	1.5	0.95	3.2	1800	441	3^b^	
	18	1.5	0.47	6.5	1800	441	4^b^	
	18	1.5	0.24	12.9	1800	441	7^b^	
	18	1.5	0.19	16.3	1800	441	7^b^	
	18	1.5	0.16	19.5	1800	441	8^b^	

^a^Mean particle diameter extracted from TEM analysis

^b^Average crystallite diameter extracted from profile refinements of powder XRD data

We hereby report on how synthetic parameters such as injection temperature and time, reaction holding time and metal to surfactant molar ratio affect and control the *ε*-Co nanoparticle size by means of the hot injection burst nucleation approach, using DCB, Co_2_(CO)_8_ and OA. Our systematic study is evaluated in view of findings reported by Iablokov et al. [18] and Ma et al. [20]. In addition, we provide recommendation for optimized production of solution-based *ε*-Co NPs in the size range ~4–10 nm. The findings are presented and discussed on the basis of DLS, ICP-OES, XRD and TEM measurements.

## Results

### Dispersions of Co NPs and synthesis yield

**Fig. 1:**
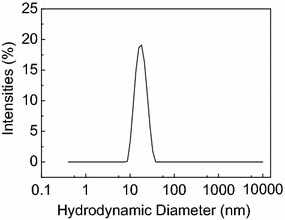
DLS measurements of dispersed *ε*-Co NPs. Hydrodynamic diameter distribution curve (*log scale*) weighted by intensity, of OA-surface coated cobalt NPs in hexane dispersion. Z-average hydrodynamic diameter = 16.9 ± 0.1 nm, as obtained from 9 replicate measurements, PDI = 0.06 ± 0.02

Diluted dispersions of OA surface coated cobalt NPs in hexane were prepared and characterized by DLS in order to determine the agglomerated state and hydrodynamic diameter of the nanoparticles. All prepared dispersions have a monomodal (only one peak) size distribution, and mean hydrodynamic diameters in the range of 13–25 nm. The hydrodynamic diameters are larger than the measured mean diameters from TEM analysis (*i.e*. 4–10 nm, see particle diameter control section below) because of the contribution of the chemisorbed surfactant (OA) on the particles surface, as well as coordinated solvent molecules. The polydispersity index (PDI) for the analysed samples was in all cases lower than 0.20, indicating near monodispersed particles [21]. A representative hydrodynamic diameter distribution curve of the Co NPs dispersions is given in Fig. 1.

DLS data for the nanoparticle dispersions collected over a time frame of 1 month did not show any indication of particle agglomeration. Therefore, the colloidal nature of the dispersions is promising with respect to subsequent deposition of free standing nanoparticles onto 2D or 3D support materials. Any agglomerated nanoparticles in suspension are likely to give aggregates of metallic NPs on the support when deposited, which is undesirable.

With applications in mind, knowledge of the exact cobalt quantity in the stable suspension is of high importance. Based on ICP-OES, the synthesis yield of Co NPs dispersions is found to be ~75 %. Timonen et al. [22], report a crystallization yield of 89 % determined by atomic absorption spectroscopy (AAS) for dispersions prior to washing. In our case, we report the yield with respect to Co NP mass in the hexane suspension *after* washing and re-dispersion, *i.e*., all sources of product loss (cobalt-OA complex formation, cobalt deposition on flask walls, on the magnetic stirrer as well as loss during washing cycles and drying steps) are reflected in the reported yield.

### Phase purity, allotropic form and unit cell dimensions of synthesized Co NPs

**Fig. 2:**
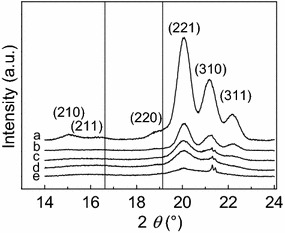
Selected powder X-ray diffraction patterns of *ε*-Co NPs. Samples were synthesized at 441 K, 5 s injection time, 1800 s reaction holding time and at $\frac{[Co]}{[OA]}$ equal to *a*) 19.5, *b*) 12.9, *c*) 6.5, and *d*) 3.2 and *e*) 2.1. Estimated average crystallite diameters: *a*) 7.6 nm, *b*) 6.9 nm, *c*) 4.1 nm, *d*) 3.4 nm and *e*) 2.2 nm. Wavelengths Mo Ka_1_ = 0.07093 nm and Ka_2_ = 0.07136 nm. Miller indices given for Bragg reflections from *ε*-Co. *Vertical lines* indicating expected positions of CoO peaks. Peak at 2*θ* = 21.3° from Si (220)

The bulk structural properties and phase purity of the synthesized *ε*-Co NPs were derived from powder XRD measurements. Diffractograms of selected samples with different crystallite sizes are presented in Fig. 2. The observed diffraction peaks with respect to positions and relative intensities correspond to *ε*-Co with the cubic *β*-Mn type structure. Miller indices are assigned to the reflections. The X-ray diffractograms of the samples with the larger cobalt particles show no indications of CoO, Fig. 2. This indicates that the pentane washing procedure for preparation of XRD specimens is sufficiently mild to prevent deep oxidation of the metallic surface. However, for the smaller particles powder XRD shows in some cases, weak indications for partial oxidation to CoO (diffractograms d, e in Fig. 2). For clarity, the expected peak positions of CoO are added in Fig. 2 as vertical lines.

**Fig. 3:**
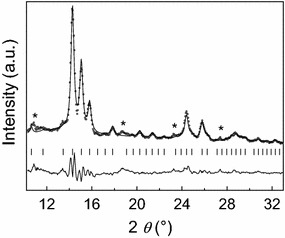
Synchrotron powder XRD intensity profiles for *ε*-Co at ambient temperature. Observed (*circles*), calculated (*upper line*), and difference profiles (*lower line*) are shown along with positions for Bragg reflections (*vertical bars*). Impurity peaks are denoted with *asterisk* (*). Wavelength = 0.050566 nm

To reveal crystallographic data for the *ε*-Co phase a selected sample was investigated by means of synchrotron powder XRD, Fig. 3. Rietveld refinements using the structural model reported by Dinega and Bawendi [10] as starting point confirmed the cubic *β*-Mn type structure (space group *P*4_1_32). Obtained unit cell parameter, *a* = 0.6098 ± 0.0003 nm, is in good agreement with the reported *a* = 0.6097 ± 0.0001 nm [10]. The synchrotron X-ray diffractogram revealed some weak additional reflections (indicated by asterisk in Fig. 3), which were not observed in the home laboratory. The origin of these reflections is not fully understood; however, possibly some can be related to hcp/ccp intergrowth particles. The refined atomic coordinates; Co(1) in 8(c) x, x, x with x = 0.062(1); Co(2) in 12(d) 1/8, y, z with y = 0.190(4) and z = 0.467(3) comply with the *β*-Mn structure.

### Particle diameter control

A series of parameters may affect the NP diameter and the size distribution in hot injection burst nucleation syntheses with OA as surfactant. In order to explore their influence on particle diameter, injection time (1–5 s), injection temperature (437–453 K), reaction holding time (300–7200 s) as well as $\frac{[Co]}{[OA]}$ molar ratio (2.1–19.5) were systematically varied.

**Fig. 4:**
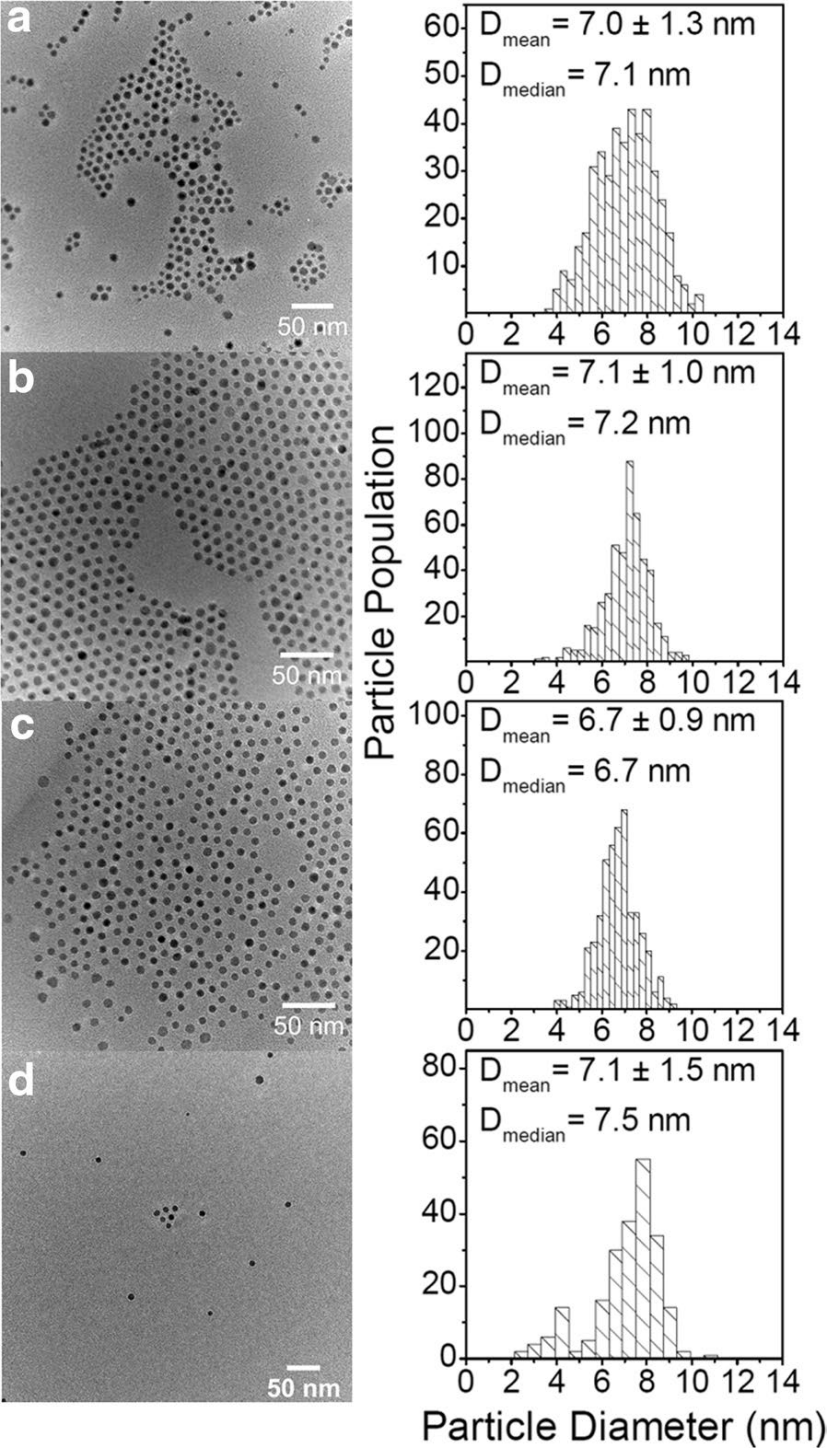
TEM images of *ε*-Co NPs from reproducibility experiments. Samples were synthesized at injection temperature 447 ± 0.5 K, $\frac{[Co]}{[OA]} = 12.9,$ injection time = 5 s, reaction holding time = 1800 s. Their corresponding particle diameter distributions were obtained from evaluating ~250–500 particles. *Scale bars* 50 nm

Prior to this parameter screening, the reproducibility of the synthesis approach was evaluated, *i.e.,* four replicate syntheses of cobalt nanoparticles were performed, with injection time 5 s, reaction holding time 1800 s, injection temperature 447 ± 0.5 K, and $\frac{[Co]}{[OA]} = 12.9$. Figure 4 presents TEM images and the particle diameter distributions from the four replicate syntheses.

The histograms, in Fig. 4, indicate that the particle diameter distributions are asymmetric, featuring a tail at lower diameters. For this reason, both mean and median particle diameters ($\bar{D}$_mean_ and $\bar{D}$_median_) are reported. Table 2 reports the 95 % bias corrected confidence intervals for both $\bar{D}$_mean_ and $\bar{D}$_median_ of the four replicas, and the corresponding values for the pooled four replicate experiments. A corresponding analysis was performed for a second series of experiments (with injection time 5 s, reaction holding time 1800 s, injection temperature 441 K and $\frac{[Co]}{[OA]} = 12.9$ (see Additional file 1). These results clearly indicate that NPs are synthesized in a reproducible and robust manner with respect to $\bar{D}$_mean_ and $\bar{D}$_median_.

**Table 2:** Bias corrected 95 % confidence intervals of mean and median particle diameters of the four replicate experiments

	$\bar{D}_{mean}$ (nm)	$\bar{D}_{median}$ (nm)
Replica 1 (Fig. 4a)	6.9–7.2	6.8–7.2
Replica 2 (Fig. 4b)	7.0–7.2	7.1–7.2
Replica 3 (Fig. 4c)	6.7–6.8	6.7–6.8
Replica 4 (Fig. 4d)	6.9–7.3	7.2–7.5
Pooled sample (Fig. 4a–d)	6.9–7.0	6.9–7.1

### Effect of injection time and reaction holding time on particle size

By changing the injection of the dissolved Co_2_(CO)_8_ into the hot round flask from slow (5 s) to fast (1 s), no significant differences on the Co NPs diameter and their size distribution were observed (see Figure in Additional file 1). The particle diameter on fast injection, $\bar{D}$_mean_ = 8.7 ± 1.5 nm (1 s), is slightly smaller than when the injection takes place slower $\bar{D}$_mean_ = 9.6 ± 1.4 nm (5 s; replica 1), 9.4 ± 1.4 nm (5 s; replica 2) and 9.4 ± 1.4 nm (5 s; replica 3).

**Fig. 5:**
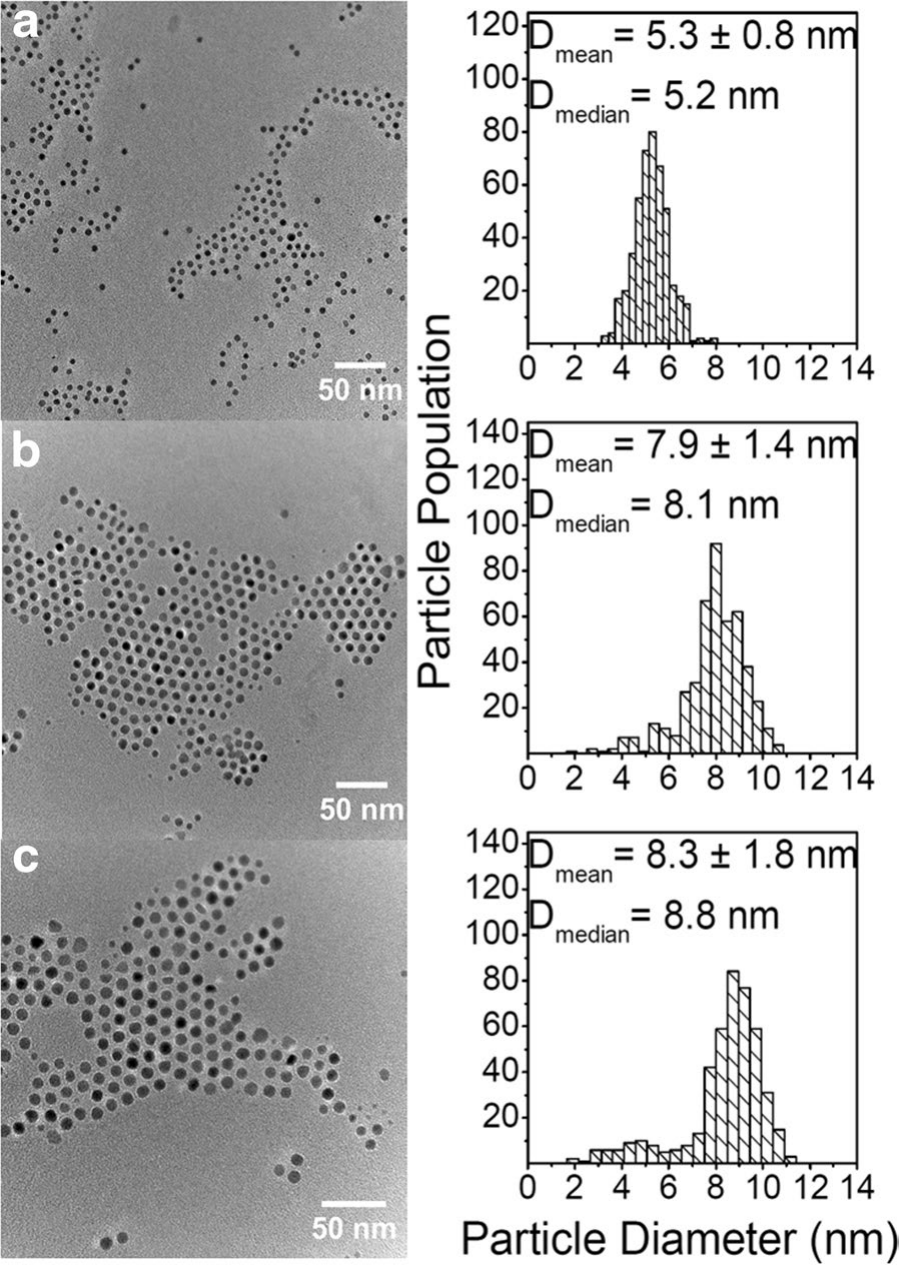
TEM images of *ε*-Co NPs synthesized varying the reaction holding time. Synthesis conditions: injection temperature = 443 K, $\frac{[Co]}{[OA]} = 12.9,$ injection time = 5 s, and reaction holding time: **a** 300, **b** 1800 and **c** 7200 s. Their corresponding particle diameter distributions were obtained from evaluation of ~500 particles. *Scale bars* 50 nm

The effect of the reaction holding time was explored by performing a time-resolved experiment where the Co NPs were synthesized under standard experimental conditions (injection time 5 s, injection temperature 443 K and $\frac{[Co]}{[OA]} = 12.9$), and small aliquots were extracted during the synthesis and cast on carbon-coated transmission electron microscopy (TEM) grids, Fig. 5. The particles undergo growth during the first 1800 s, followed by a stage giving significantly broadening of the size distribution (as reflected in σ) during particle aging (7200 s) without any significant increase in $\bar{D}$_mean_. At reaction holding times of 1800 and 7200 s Fig. 5b, c, the shape of the size distribution is asymmetric and falls into the category of left-skewed, where, $\bar{D}$_median_ is larger than $\bar{D}$_mean_, featuring a tail at the low-diameter side. This is not observed at short reaction holding times (Fig. 5a). In conclusion, a more narrow size distribution of Co NPs can be obtained by using shorter reaction holding times. It should also be mentioned that left-skewed histograms do not only correlate with injection time, as demonstrated in the Additional file 1. The asymmetric particle diameter distributions currently observed at long reaction holding times, may reveal information on the growth mechanism of the as-synthesized nanoparticles [23].

### Effect of injection temperature on particle size

**Fig. 6:**
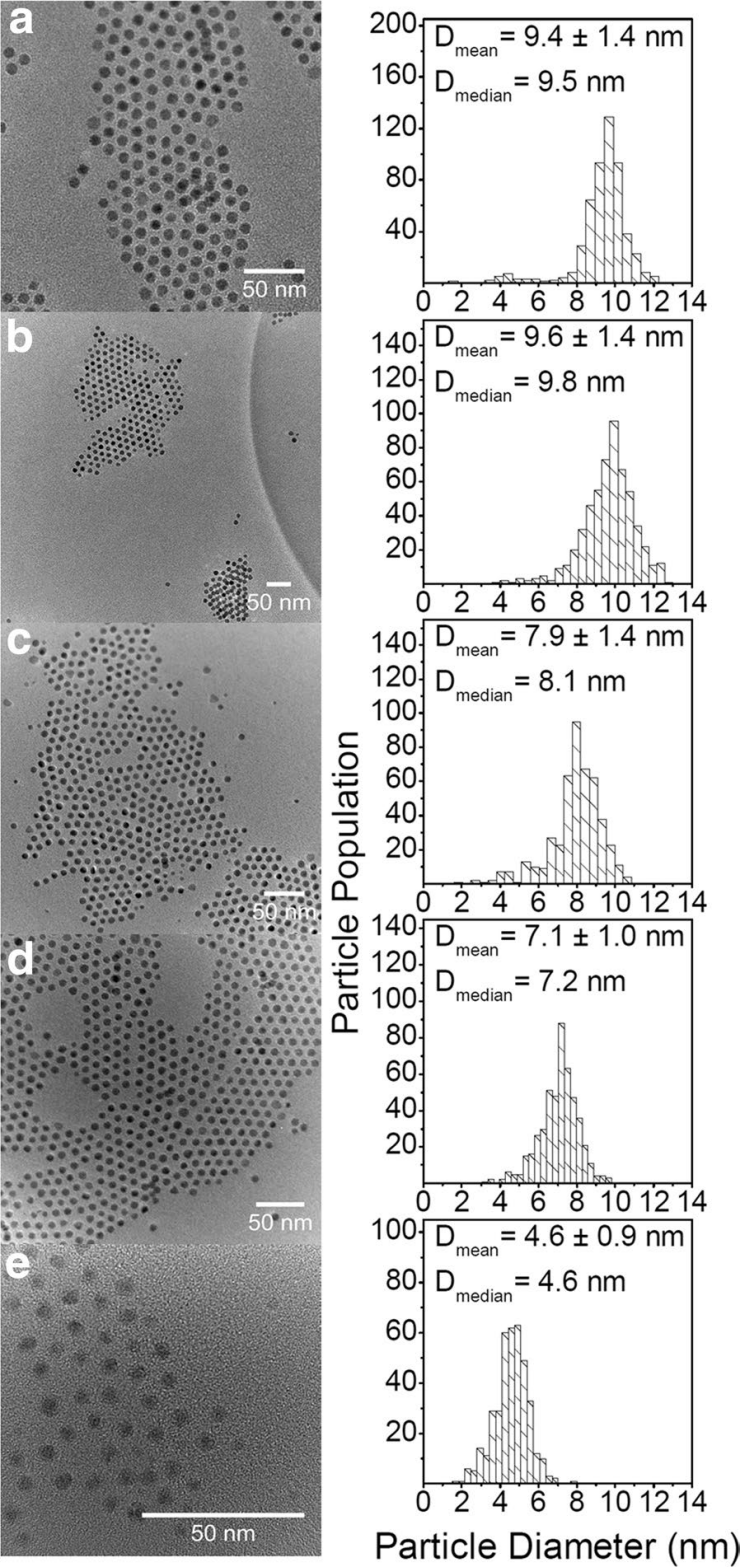
TEM images of *ε*-Co NPs synthesized varying the injection temperature. Synthesis conditions: $\frac{[Co]}{[OA]} = 12.9,$ injection time = 5 s, reaction holding time = 1800 s at **a** 437, **b** 441, **c** 443, **d** 447 and **e** 452 K, along with their corresponding particle diameter distributions obtained from evaluation of ~500 particles. *Scale bars* 50 nm

In the study of the effect of injection temperature on the particle diameter of the *ε*-Co NPs, other parameters were fixed: reaction holding time (1800 s), molar ratio of cobalt to surfactant ($\frac{[Co]}{[OA]} = 12.9$) and injection time (5 s). The syntheses were performed in the temperature range of 437–452 K. Representative TEM images of Co NPs produced at 437, 441, 443, 447 and 452 K are shown in Fig. 6 along with their corresponding particle diameter distributions.

**Fig. 7:**
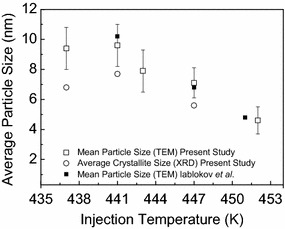
Comparison of average diameters of *ε*-Co NPs obtained at different injection temperatures. Synthesis conditions: $\frac{[Co]}{[OA]} = 12.9,$ injection time = 5 s and reaction holding time = 1800 s. *Open circles* , as extracted from powder XRD patters; *open squares*, $\bar{D} \pm \sigma$ from TEM analysis and *solid squares*, as reported from TEM analysis by Iablokov et al. [18]

The particle diameter is decreasing when the injection temperature is increased, see Figs. 6 and 7. The upper temperature limit of the synthesis (452–453 K) is defined by the boiling point of the solvent DCB (*T*_b_ = 453.5 K). It appears that there exists a lower temperature limit of around 441 K, below which no variation in particle diameter is observed (Fig. 6a, b). The observed trend is in good agreement with Iablokov et al. [18] (see Fig. 7), although achieved at a different $\frac{[Co]}{[OA]}$ molar ratio. However, when comparing with the work of Iablokov et al. [18], our results indicate an inferior size distribution (RSD = 14–20 %) in accordance with Puntes et al. [19]. The results prove that particle diameter can be tuned and controlled by varying the temperature of the hot OA-DCB solution.

The TEM analysis of ~500 NPs for extracting the particle diameter is laborious. Therefore, it was evaluated whether data on average crystallite diameter could be estimated by XRD as a supplementary or alternative approach. Figure 7 compares the derived average crystallite and particle diameters as estimated from XRD and TEM, respectively. The agreement is fairly good; however XRD predicts systematically slightly smaller diameters than TEM, which is reasonable in view of possible partial cobalt oxidation as well as the particles observed by TEM not necessarily being single crystallite, see discussion section.

### Effect of oleic acid (OA) concentration

**Fig. 8:**
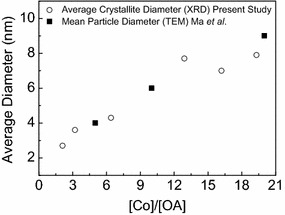
Average particle diameters obtained for *ε*-Co NPs as a function of $\frac{[Co]}{[OA]}$. Synthesis conditions: injection temperature 441 K, injection time 5 s, reaction holding time 1800 s. *Open circles* show the average crystallite diameters, as extracted from XRD analysis, of *ε*-Co NPs synthesized in this work. *Solid squares* represent the mean diameters from TEM analysis of *ε*-Co NPs reported by Ma et al. [20]. Relevant XRD patterns are given in Fig. 2

In the study of the effect of the oleic acid concentration on *ε*-Co NP size, the amount of Co_2_(CO)_8_ was fixed (0.52 g), whereas, the OA concentration was adjusted to cover the $\frac{[Co]}{[OA]}$ range from 2.1 to 19.5. Furthermore, the reaction holding time (1800 s), injection temperature (441 K) and injection time (5 s) were fixed. XRD was used to extract data on the crystallite diameter. Figure 8 presents the estimated average crystallite diameters of the derived *ε*-Co NP as a function of the $\frac{[Co]}{[OA]}$ molar ratio.

According to the XRD analysis, an increased $\frac{[Co]}{[OA]}$ molar ratio from 2.1 to 12.9 has a pronounced effect on the average cobalt NPs crystallite diameter, which increases from 2 to 8 nm (Fig. 8). However, any further increase of $\frac{[Co]}{[OA]}$ to 16.3 and 19.5 did not result in larger crystallites. This indicates that an average crystallite diameter of 8 nm is the upper size limit for the current approach. Note that it is likely that TEM would give slightly larger diameter values; see above and Fig. 7. Our findings follow the same trend as reported by Ma et al. [20] (reported data in [20] extracted from TEM analysis). In conclusion, the average cobalt crystallite diameter is decreasing when the cobalt to surfactant molar ratio is reduced.

**Fig. 9:**
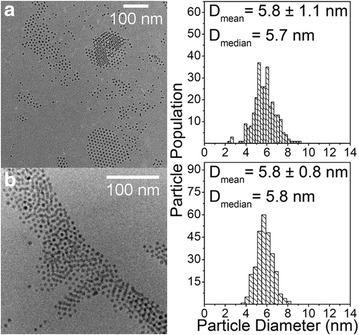
TEM images of *ε*-Co NPs synthesized at $\frac{[Co]}{[OA]} = 6.5$. Samples are synthesized at **a** 441 and **b** 446 K, injection time = 5 s, reaction holding time = 1800 s. Their corresponding particle diameter distributions are obtained from counting ~300 particles. *Scale bars* 100 nm

As described above (effect of injection temperature on particle size section), Iablokov et al. [18] observed the same particle diameter trend as we report in this study, when using injection temperature as the tuning parameter (Fig. 7). However, they applied a lower $\frac{[Co]}{[OA]}$ molar ratio (6.5) than currently (12.9). Additional syntheses were therefore carried out for $\frac{[Co]}{[OA]} = 6.5$ in steps of ~2 K in the range 441–450 K. Representative TEM data are shown in Fig. 9, with obtained particle diameters of 5.8 ± 1.1 nm (441 K) and 5.8 ± 0.8 nm (446 K). For injection temperatures close to the boiling point of the solvent, particles in the 3–4 nm size range were obtained (data not shown). Hence, for a fixed $\frac{[Co]}{[OA]} = 6.5,$ variation of injection temperature is not a mean for tuning the particle diameter over a large range of sizes. We observe that the particle size becomes quite insensitive to variations in injection temperature for $\frac{[Co]}{[OA]} < 12.9$.

## Discussion

A variety of solvent-surfactant combinations provide *ε*-Co nanoparticles when using the hot injection approach and Co_2_(CO)_8_ as cobalt precursor [14, 17]. Just a handful of these concern the DCB-OA solvent-surfactant combination (Table 1) [18–20], which is the target for the current systematic study of reaction parameters controlling the diameter of dispersed Co NPs. We show that the mean particle diameter can be reproducibly controlled between 4 and 10 nm (with RSD ~14–20 %) by either tuning the injection temperature, or the $\frac{[Co]}{[OA]}$ molar ratio. Reaction holding time and injection time have less influence on the investigated conditions. The syntheses yield for washed and redispersed nanoparticles is ~75 % and stable dispersions are formed in hexane. The smaller particles (< 3–4 nm) may suffer from partial or full oxidation to CoO. Such undesired oxidation is best suppressed at a low $\frac{[Co]}{[OA]}$ molar ratio and moderate injection temperatures. OA is a capping agent forming strong covalent bonding [1] to cobalt and prevents deep oxidation as well as major particle growth. A high OA concentration affects the particle growth to such an extent that it cancels out the influence of injection temperature on the NP size.

We show that reproducible syntheses can only be achieved when strictly controlling the two key size determining parameters, injection temperature and $\frac{[Co]}{[OA]}$ molar ratio, as well as, suitably selecting the less sensitive parameters to reasonable values such as injection time and reaction holding time. Good reproducibility required the use of an identical apparatus, *i.e.,* same glass ware, heating and isolation system, location of thermocouple etc. Although, studies by Ma et al. [20] and Iablokov et al. [18] also report particle diameters in the range 4–10 nm (Table 1), there are discrepancies in the applied conditions and in the resulting NP size. It is tempting to suggest that the dissimilarity in data between Ma et al. [20] and our study, has its origins in technical factors. Possibly, poor temperature control in the synthesis apparatus of Ma et al. [20], would explain the discrepancy in reported injection temperature for certain particle sizes as function of $\frac{[Co]}{[OA]}$. Furthermore, poor temperature control in the synthesis apparatus would also explain why the injection temperature used by Ma et al. [20] (463 K) is higher than the boiling point of the solvent (453.5 K). It remains open why Iablokov et al. [18] were able to obtain NPs over the diameter range ~4–10 nm at $\frac{[Co]}{[OA]} = 6.5$, at which conditions we constantly produced small particles within a narrow size range.

In comparison with TEM imaging and data analysis of particle diameter and size distribution, a corresponding XRD analysis is fast and integrated with phase content analysis. Whereas the estimated crystallite diameter from XRD represents the volume average of the exposed sample (some mg), the TEM data for the mean (or median) particle diameter refers to the diameter projected value for a limited number of particles (~500). However, the average crystallite diameter as determined by XRD is underestimated, unless the applied model takes into account stress, stacking disorder, chemical heterogeneities etc. Furthermore, crystallite sizes extracted from XRD can only be fully compared with single crystal particle diameters obtained from TEM. In our case, the adopted XRD approach systematically underestimated the average *ε*-Co NP diameter relative to TEM, see Fig. 7. We indeed believe this can be explained by the fact that the particles are not single crystallites. In addition, the cobalt NPs may also have suffered from partial oxidation, giving rise to a thin CoO shell. A thin cobalt oxide layer on the Co NP will give a larger TEM particle size. Despite these facts, XRD appears an excellent tool for a fast evaluation of crystallite diameter (which in turn gives indirect information on particle size) in the screening of synthesis parameters.

The histogram size distribution may contain key data for assessing the particle growth mechanism [23]. We note that several histograms possess asymmetric distributions (see Figs. 5, 6). Particle growth proceeds via Ostwald ripening and/or coalescence. If the main growth mechanism is coalescence (merging of nanoparticles), log-normal distributions are expected [24]. On the other hand, if Ostwald ripening is dominant (larger particles grow at the expense of smaller ones), the size distribution is expected to have a bias toward larger particle diameters. The asymmetric particle diameter distributions currently observed might indicate Ostwald ripening. However, careful investigations should be carried out allowing the particles to grow to even larger sizes so that any history of the initial distribution is lost [24].

## Conclusions

In summary, careful control of the reaction conditions in the hot injection decomposition of a Co_2_(CO)_8_ precursor in the presence of oleic acid (OA) can yield in a reproducible manner, *ε*-Co NPs with a narrow size distribution over the 4–10 nm size range. We have demonstrated that the obtained particle sizes can be varied significantly by controlling either the metal to surfactant molar ratio, or the injection temperature. By increasing the metal to surfactant molar ratio the mean particle diameter of the *ε*-Co NPs has been found to increase. Furthermore, an increase of the injection temperature results in a decrease in the mean particle diameter of the *ε*-Co NPs, when the metal to surfactant molar ratio $\left( {\frac{[Co]}{[OA]}} \right)$ is fixed at ~ 12.9. Additionally, our experimental data indicated that particle size becomes insensitive to variations in injection temperatures for $\frac{[Co]}{[OA]} < 12.9$. Ultimately, while variations of the injection time of the cobalt precursor into the hot OA-DCB solution gave insignificant differences on the measured Co NPs diameters and size distributions, it was experimentally demonstrated that a more narrow size distribution of *ε*-Co NPs can be obtained by using shorter reaction holding times.

## Perspectives

For the preparation of cobalt based metal-on-support model catalysts with specific metal loading and good metal dispersion as outlined by An and Somorjai [8], careful control of particle diameter, particle concentration and any presence of non-agglomerated particles are crucial. Currently reported procedure for preparation of Co NPs as colloidal dispersions fulfils these criteria. A desirable next step is to expand the synthesis recipes to include a second metal for forming bimetallic particles; for instances by including Pt or Re [7] as these are common promoters in Co-based FT catalysts. Additional procedures are needed with respect to depositing the particles on suited support materials (Al_2_O_3_ based), removal of surfactants, activation of the catalysts without hampering the original narrow size distribution and NP morphology. Such efforts will result in high quality model catalysts suited for single parameter studies.

## Methods

### Chemicals

Dicobalt octacarbonyl [Co_2_(CO)_8_ in hexane vapor, ≥90 % Co], oleic acid [CH_3_(CH_2_)_7_CH = CH(CH_2_)_7_COOH, OA, ≥99 %], 1,2-dichlorobenzene (C_6_H_4_Cl_2_, DCB, 99 %, anhydrous), 2-propanol (CH_3_CHOHCH_3_, 99.5 %, anhydrous), hexane (C_6_H_14_, 95 %, anhydrous) and pentane (C_5_H_12_, 98 %) from Sigma-Aldrich were used without further purification. Co_2_(CO)_8_ and OA were stored under Ar atmosphere at 278 and 253 K, respectively.

### Nanoparticle synthesis

All syntheses were carried out employing standard Schlenk- and glovebox techniques in Ar atmosphere (5 N). Typically a 250 mL four-neck Pyrex flask equipped with high resistance silicone septa (Versilic) and inlet for Ar on two of the side arms was used. The reaction temperature was monitored with a K-type thermocouple protected in a quartz liner on the third side neck, and the temperature profiles were logged using a Fluke thermometer (model 53/54 II B). Effluent was sent to the ventilation system via an Allihn condenser (400 mm) connected to a bubbler containing 0.4 M KMnO_4_ for CO abatement. The reaction mixture and the Huber Siloil (high temperature) bath were continuously stirred with magnetic bars at 800 rpm (revolutions per minute).

*ε*-Co NPs were obtained by thermal decomposition of Co_2_(CO)_8_ when rapidly injected into a hot solution of DCB containing dissolved OA. In a typical synthesis, 50–380 μL OA was dissolved in 15 mL DCB under Ar flow. The solution was subsequently heated to the targeted injection temperature (437–452 K) under stirring. In the meantime, a precursor solution of 0.52 g Co_2_(CO)_8_ was dissolved in 3 mL DCB in a glove box and sealed in an airtight vial. When the DCB-OA mixture reached the targeted temperature, the precursor solution was withdrawn into a syringe (G 20 needle) and injected into the four neck flask within an injection time of 5 s. Thermal decomposition of Co_2_(CO)_8_ into Co metal and CO is extremely rapid at the target temperatures as Co_2_(CO)_8_ decomposes already below 363 K under inert atmosphere [25], evidenced by a short burst of CO evolution and formation of a black colloidal solution. The solution temperature drops some 15–20 K at the injection of the cobalt precursor, due to the endothermic nature of the decomposition reaction as well as the addition of cold solvent. Heating was maintained after the injection and the temperature climbed back to the initial target value within 60–180 s. The obtained colloidal suspension was aged for a specific time (300–7200 s) and subsequently quenched by adding 15 mL of cold DCB. Thereafter 2-propanol was added to flocculate the particles. The solution was centrifuged at 4000 rpm (G-force 1667) for 300 s. The supernatant was discarded and the precipitate underwent the aforementioned washing cycle for at least three more times. The supernatant was typically clear and colorless, indicating complete reaction and complete precipitation. Any observation of a clear blue colored supernatant would have indicated the presence of cobalt-oleate complexes [26]. The washed precipitate was subsequently redispersed in hexane and 50 μL of OA was added to protect the as-synthesized NPs from oxidation. At the end of the synthesis, ~4–10 nm *ε*-Co NPs coated with OA were produced.

### Characterization

NPs and suspensions of dispersed NPs were characterized by inductively coupled plasma optical emission spectroscopy (ICP-OES), dynamic light scattering (DLS), powder X-ray diffraction (XRD), synchrotron powder XRD and transmission electron microscopy (TEM).

ICP-OES was performed by Molab A.S. on dried Co NP powders originating from stable hexane dispersions. Prior to analysis the Co NPs were dissolved in a mixture of nitric acid and hydrogen peroxide. The synthesis yield is defined as the mass of cobalt product present in the hexane suspension after at least three washing cycles, divided by the mass of cobalt added to the synthesis via the injected Co_2_(CO)_8_ solution.

DLS data was measured on a Malvern Instruments Zetasizer-Nano ZS equipped with a 4nW He–Ne laser operating at a wavelength of 633 nm and an avalanche photodiode (APD) detector. The scattered light was measured at an angle of 173°. Cobalt NPs [~0.1 mg/mL, refractive index (n) = 2.26] dispersed in hexane [n = 1.38 and viscosity (η) = 297 μPa s] were analyzed at 298 K in a quartz cuvette (PCS1115) after filtering through 0.45 μm filters (Millex-HV, PVDF membrane). Data were recorded based on six or more replicate measurements.

Powder XRD patterns for analysis of phase purity, unit cell dimensions and crystallite size estimations were acquired in reflection geometry on a Bruker D8 Advance diffractometer with focusing Göbel mirror and Lynx Eye XE detector adapted for high energy, using Mo-K_α_ radiation (Ka_1_ = 0.07093 nm and Ka_2_ = 0.07136 nm). Powder samples of Co NPs agglomerates were obtained after additional flocculation using 2-propanol, followed by centrifugation at 9000 rpm (G-force 8437) for 300 s and a final washing with small amounts of pentane. The samples were deposited on specially cut Si-single crystal holders. Analysis of the diffraction data was performed using the TOPAS [27] and Bruker AXS DIFFRAC.EVA [28] software packages. Peak position corrections were done using NIST silicon powder (SRM 640d, *a* = 0.543123 ± 0.000008 nm) as internal standard. For crystallite size estimations, the simple Scherrer approach was not possible due to major peak overlap. TOPAS was therefore used for convolution-based profile fitting (Fundamental Parameters Approach) and determination of crystallite size. The fundamental parameters peak shape was based on the measured goniometer radii and corrected for peak asymmetry using the simple axial model. Peak broadening was modelled using a Lorentzian crystallite size term. Full width at half maximum based volume-weighted mean column height values (L _Vol_-FWHM) of coherently diffracting spherical domains (k = 0.89) are reported as average crystallite diameters.

High resolution synchrotron powder XRD data were collected at the Swiss-Norwegian Beamline (SNBL) BM01B at the European Synchrotron Radiation Facility (ESRF), Grenoble, France. The sample was filled in 1.0 mm capillary and rotated during data collection. The zero point and wavelength (*λ* = 0.050566 nm) was determined using a Si NIST standard. Rietveld refinements were done using the FullProf Suite of programs [29]. The measured data were rebinned into steps of 0.05°. Altogether 570 data points and 64 Bragg reflections were used in the refinements. One unit cell parameter, three atomic coordinates, one isotropic displacement factor and up to four profile parameters were refined. The background was determined by interpolation between 14 data points. Obtained R_Bragg_ = 11.3, R_p_ = 6.92 whereas R_expected_ = 2.85.

Transmission electron microscopy (TEM) images were acquired by means of a JEOL JEM-2100F microscope operating at 200 kV, equipped with a Gatan Orius SC 200D 2, 14-bit, 11-megapixel CCD and a spherical aberration corrector in the objective lens. All the samples for TEM analysis were prepared by drop casting 20 μL of the relevant NP-suspension onto carbon-coated 300 mesh, 3 mm copper grids, Agar Scientific UK, and drying under inert atmosphere.

### Histograms for particle diameter distribution and statistical analysis

The histograms of the NPs were obtained by measuring the diameter of 250–500 NPs using ImageJ [30], assuming the particles to be spherical. As the distribution of the particle diameters may be asymmetric, both $\bar{D}$_mean_ and $\bar{D}$_median_ values are reported. In addition, we report the relative standard deviation $({\text{RSD}}) = \frac{\sigma }{{\bar{D}_{mean} }} \times 100\,\%$, where σ is the standard deviation and $\bar{D}$_mean_ is the sample mean.

95 % bias corrected confidence intervals (CIs) were calculated for the obtained mean and median particle diameters of NP syntheses that had been performed with three or four replicas. A non-parametric approach was selected due to the expected non-normal distribution of the  measured diameters. Instead of making any prior assumptions of the size distribution, bootstrapping was chosen to calculate the CIs [31, 32].

## Additional file

10.1186/s13065-016-0156-1 TEM and statistical analysis.pdf. TEM images of cobalt nanoparticles synthesized at 441 ± 1 K, reaction holding time = 1800 s and $\frac{[Co]}{[OA]} = 12.9,$ where the injection time was varied.

## References

[1] Sun S, Murray CB (1999). Synthesis of monodisperse cobalt nanocrystals and their assembly into magnetic superlattices. J Appl Phys.

[2] Murray CB, Kagan CR, Bawendi MG (2000). Synthesis and characterization of monodisperse nanocrystals and close-packed nanocrystal assemblies. Annu Rev Mater Sci.

[3] Yang Z, Lisiecki I, Walls M, Pileni M-P (2013). Nanocrystallinity and the ordering of nanoparticles in two-dimensional superlattices: controlled formation of either core/shell (Co/CoO) or hollow CoO nanocrystals. ACS Nano.

[4] Puntes VF, Krishnan KM (2001). Synthesis, structural order and magnetic behavior of self-assembled and ε-Co nanocrystal arrays. IEEE Trans Magn.

[5] Herranz T, Deng X, Cabot A, Guo J, Salmeron M (2009). Influence of the cobalt particle size in the CO hydrogenation reaction studied by in situ X-ray absorption spectroscopy. J Phys Chem B.

[6] Morales F, Weckhuysen BM, Spivey JJ, Dooley KM (2006). Promotion effects in Co-based Fischer-Tropsch catalysis. Catalysis.

[7] Rytter E, Skagseth TH, Eri S, Sjåstad AO (2010). Cobalt Fischer–Tropsch catalysts using nickel promoter as a rhenium substitute to suppress deactivation. Ind Eng Chem Res.

[8] An K, Somorjai GA (2012). Size and shape control of metal nanoparticles for reaction selectivity in catalysis. ChemCatChem.

[9] Cavalier M, Walls M, Lisiecki I, Pileni M-P (2011). How can the nanocrystallinity of 7 nm spherical Co nanoparticles dispersed in solution be improved?. Langmuir.

[10] Dinega DP, Bawendi MG (1999). A solution-phase chemical approach to a new crystal structure of cobalt. Angew Chem Int Ed.

[11] Ram S (2001). Allotropic phase transformations in HCP, FCC and BCC metastable structures in Co-nanoparticles. Mater Sci Eng A.

[12] Westgren A, Phragmen G (1925). Zum kristallbau des Mangans. Z f Physik.

[13] Yang HT, Shen CM, Wang YG, Su YK, Yang TZ, Gao HJ (2004). Stable cobalt nanoparticles passivated with oleic acid and triphenylphosphine. Nanotechnology.

[14] Hyeon T (2003). Chemical synthesis of magnetic nanoparticles. ChemComm.

[15] Puntes VF, Krishnan KM, Alivisatos AP (2001). Colloidal nanocrystal shape and size control: the case of cobalt. Science.

[16] LaMer VK, Dinegar RH (1950). Theory, production and mechanism of formation of monodispersed hydrosols. J Am Chem Soc.

[17] Green M: Organometallic based strategies for metal nanocrystal synthesis. Chem Comm. 2005:3002–3011 10.1039/b501835h 15959567

[18] Iablokov V, Beaumont SK, Alayoglu S, Pushkarev VV, Specht C, Gao J, Alivisatos AP, Kruse N, Somorjai GA (2012). Size-controlled model Co nanoparticle catalysts for CO_2_ hydrogenation: synthesis, characterization, and catalytic reactions. Nano Lett.

[19] Puntes VF, Zanchet D, Erdonmez CK, Alivisatos AP (2002). Synthesis of hcp-Co nanodisks. J Am Chem Soc.

[20] Ma W-W, Yang Y, Chong C-T, Eggeman A, Piramanayagam SN, Zhou T-J, Song T, Wang J-P (2004). Synthesis and magnetic behavior of self-assembled Co nanorods and nanoballs. J Appl Phys.

[21] Kaszuba DM: Malverns’ Zetasizer customer training course. 2014

[22] Timonen JVI, Seppälä ET, Ikkala O, Ras RHA (2011). From hot-injection synthesis to heating-up synthesis of cobalt nanoparticles: observation of kinetically controllable nucleation. Angew Chem Int Ed.

[23] Granqvist CG, Buhrman RA (1976). Size distributions for supported metal catalysts: coalescence growth versus ostwald ripening. J Catal.

[24] Chen H, Yu Y, Xin HL, Newton KA, Holtz ME, Wang D, Muller DA, Abruña HD, DiSalvo FJ (2013). Coalescence in the thermal annealing of nanoparticles: an in situ STEM study of the growth mechanisms of ordered Pt–Fe nanoparticles in a KCl matrix. Chem Mater.

[25] Tannenbaum R (1994). Thermal decomposition of cobalt carbonyl complexes in viscous media. Inorg Chim Acta.

[26] Hyeon T, Chung Y, Park J, Lee SS, Kim Y-W, Park BH (2002). Synthesis of highly crystalline and monodisperse cobalt ferrite nanocrystals. J Phys Chem B.

[27] Coelho AA (2007). TOPAS Academic Version 4.1.

[28] DIFFRAC.EVA. Version 3.0 edn. Karlsruhe: Bruker AXS; 2012

[29] Rodríguez-Carvajal C (1993). Recent advances in magnetic structure determination by neutron powder diffraction. Phys B (Amsterdam, Neth).

[30] Schneider CA, Rasband WS, Eliceiri KW (2012). NIH Image to ImageJ: 25 years of image analysis. Nat Methods.

[31] Davison AC, Hinkley DV (1997). Bootstrap methods and their applications.

[32] Efron B (1987). Better bootstrap confidence intervals. J Am Stat Assoc.

