# Supplementary material for: Burst nucleation by hot injection for size controlled synthesis of ε-cobalt nanoparticles
Source: Chem Cent J. 2016 Mar 8;10:10. doi: 10.1186/s13065-016-0156-1 (PMC4782310; doi:10.1186/s13065-016-0156-1)
Supplement: Supplementary file 1 — 10.1186/s13065-016-0156-1 TEM and statistical analysis.pdf. TEM images of cobalt nanoparticles synthesized at 441 ± 1 K, reaction holding time = 1800 s and $\frac{[Co]}{[OA]} = 12.9,$[Co][OA]=12.9, where the injection time was varied. [file 13065_2016_156_MOESM1_ESM.docx]

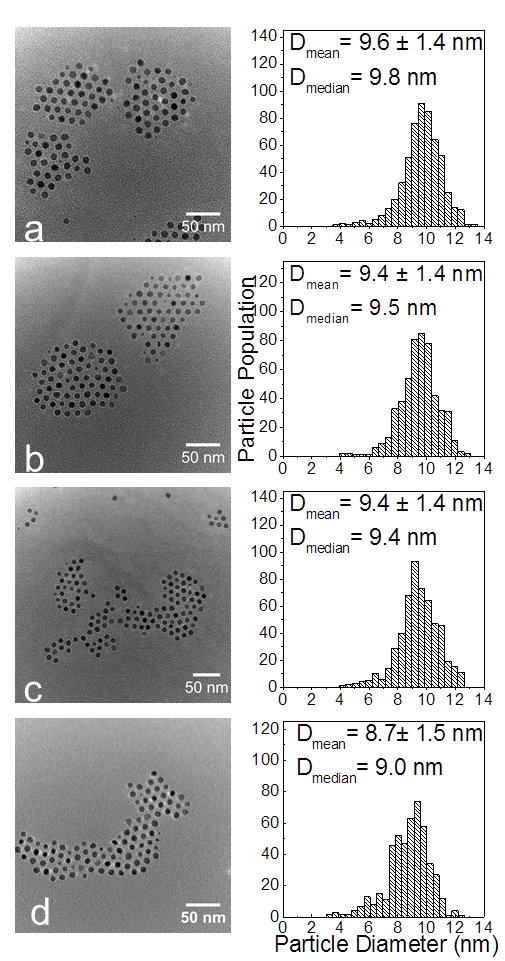


**Figure 1.** TEM images of cobalt nanoparticles synthesized at 441 ± 1 K, reaction holding time = 1800 secs and $\frac{[Co]}{[OA]}=$ 12.9, where the injection time was varied: a, b, c) three replicate synthesis with 5 secs injection time and d) 1 sec injection time. The corresponding particle diameter distributions were obtained from evaluation of ~500 particles. Scale bars 50 nm.

**Table 1** Bias corrected 95 % confidence intervals of mean and median particle diameters of the four replicate experiments presented in Figure.1 a-c, including the pooled sample median and mean values. Injection temperature 441 ± 1 K, $\frac{[Co]}{[OA]}=$ 12.9, injection time = 5 secs, reaction holding time = 1800 secs.

|  | $\bar{D}$_mean_ | $\bar{D}$_median_ |
| --- | --- | --- |
| Replica 1 (Figure 1a) | 9.5 - 9.8 | 9.5 - 9.8 |
| Replica 2 (Figure 1b) | 9.3 - 9.6 | 9.3 - 9.6 |
| Replica 3 (Figure 1c) | 9.3 - 9.5 | 9.4 - 9.6 |
| Pooled sample (Figures 1a-c) | 9.4 - 9.6 | 9.5 - 9.6 |
